# *Escherichia coli* adhesion portion FimH polarizes M2 macrophages to M1 macrophages in tumor microenvironment via toll-like receptor 4

**DOI:** 10.3389/fimmu.2023.1213467

**Published:** 2023-09-01

**Authors:** Wei Zhang, Li Xu, Xiaoyan Zhang, Jianqing Xu, Jun-O Jin

**Affiliations:** ^1^ Shanghai Public Health Clinical Center, Shanghai Medical College, Fudan University, Shanghai, China; ^2^ The Laboratory for Immunotherapy, Clinical Center for BioTherapy, Zhongshan Hospital, Fudan University, Shanghai, China; ^3^ Dpartment of Microbiology, University of Ulsan College of Medicine, ASAN Medical Center, Seoul, Republic of Korea

**Keywords:** fimH, macrophage polarization, toll-like receptor 4, myeloid differentiation factor 2, tumor-associated macrophage

## Abstract

**Background:**

Macrophages are key effector cells of innate immunity and play a critical role in the immune balance of disease pathogenesis, especially in the tumor microenvironment. In previous studies, we showed that FimH, an Escherichia coli adhesion portion, promoted dendritic cell activation. However, the effect of FimH in macrophage polarization has yet to be fully examined. In this study, we investigated the potential effect of FimH on macrophages, as well as the polarization from M2 to M1 macrophages, contributing to the overall antitumor effect

**Methods:**

Mouse bone marrow derived macrophages and peritoneal macrophages were generated to test the effect of FimH in vitro. The expression of costimulatory molecules and production of cytokines were analyzed. The effect of FimH in the tumor-associated macrophages was examine in the B16F10-tumor bearing C57BL/6.

**Results:**

FimH was found to promote M1 macrophage activation. In addition, FimH polarized M2 macrophages, which were induced by interleukin (IL)-4 and IL-13 into M1 macrophages were dependent on toll-like receptor 4 and myeloid differentiation factor 2. Moreover, FimH reprogramed the tumor-associated macrophage (TAM) into M1 macrophages in B16 melanoma tumor-bearing mice and promoted an inflammatory reaction in the tumor microenvironment (TME). Furthermore, FimH promoted M1 macrophage activation, as well as the reversion of M2 macrophages into M1 macrophages in humans. Finally, FimH treatment was found to enhance the anti-cancer immunity of anti-PD-L1 antibody by the induction of M1 polarization from TAM.

**Conclusion:**

This study demonstrated the potential effect of FimH on the activation of macrophages, responsible for the repolarization of M2 macrophages into the M1 phenotype via the TLR4 signaling pathway. Moreover, FimH could also reprogram TAM polarization to the M1 status in the TME, as well as enhance the anti-tumor activity of immune checkpoint blockade.

## Graphical abstract

FimH altered macrophage polarization in the tumor microenvironment.
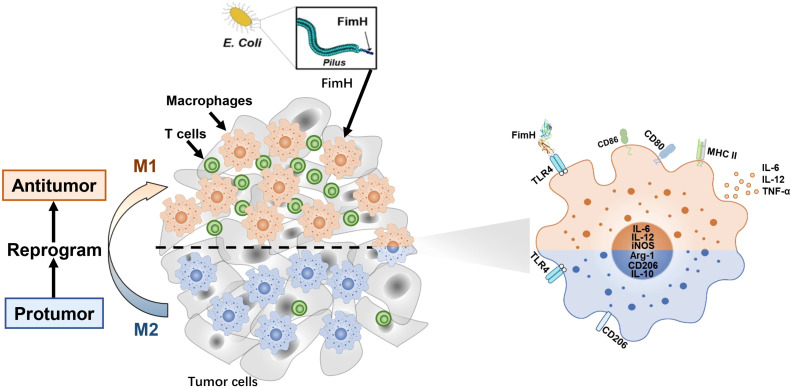

## 1 Introduction

As the key effector cells of innate immunity and antigen-presenting cells, macrophages exert strong phagocytic activity against bacteria and play a vital role in the immune system (1, 2). Macrophages can be polarized into phenotypes with different functions under the action of different cytokines or pathogenic signals (3, 4). Macrophages can be divided into M1 and M2 types. M1 macrophages, or classically activated macrophages, defend against bacterial infection and tumor cells and are involved in tissue inflammation (3). On the other hand, M2 macrophages, or alternative activated macrophages, play a role in suppressing inflammation and regenerating tissues, as well as tumor growth, via the secretion of anti-inflammatory cytokines (5). The polarity of these macrophages can be displayed by cytokines or immune stimulators. M1 macrophages can be polarized with the stimulation of lipopolysaccharide (LPS) or interferon (IFN)-γ, whereas M2 macrophages can be polarized by the stimulation of interleukin (IL)-4 and IL-13 (6). M1 and M2 macrophages differ in terms of membrane receptor expression, effector function, and cytokine production. M1 macrophages can be defined by the expression of CD80, CD86, and major histocompatibility complex (MHC) class II with the secretion of IL-12 and tumor necrosis factor (TNF)-α (7). High levels of inducible nitric oxide synthase (iNOS) are needed by M1 macrophages for NO synthesis (8). In contrast, M2 macrophages highly express CD206 and arginase 1 (Arg1) with the production of IL-10 after polarization (6).

The tumor microenvironment (TME) comprises non-cancerous host cells and non-cellular components and plays an important role in various aspects of tumorigenesis (9, 10). Among them, some immune cells affect TME establishment and tumor growth. Tumor-associated macrophages (TAMs), the most abundant immune cells in the TME, show strong plasticity in different tumor sites and stages (11, 12). TAMs are induced to form M2 macrophages via local mediators and support tumor immune escape, angiogenesis, and matrix remodeling (13). Recent advances in cancer immunotherapy, such as cancer vaccine, immune checkpoint blockade, and adoptive cell transfer, have mainly focused on the killing ability of cytotoxic T lymphocytes (CTLs) in the elimination of tumor cells (14–16). However, CTL-based immunotherapy shows limitations in the treatment of solid tumors due to the presence of immunosuppressed TAMs (17, 18). Therefore, in order to improve the efficiency of immunotherapy, there is a need to fully understand TAM, including the mechanism underlying how the activation of macrophages from TAMs to M1 macrophages can be reversed.

FimH, the adhesion portion of type I fimbriae, is a highly conserved protein located on the surface of *Escherichia coli* (*E. coli*) (19). As a toll-like receptor 4 (TLR4) ligand, FimH can stimulate and induce the activation of natural killer (NK) cells and dendritic cells (DC) through the TLR4 signaling pathway (20, 21). In the case of well-defined TLR4 ligand, LPS requires myeloid differentiation factor 2 (MD2) to stimulate TLR4, while FimH can directly bind and stimulate TLR4 without MD2 (22). In addition, in previous studies we found that FimH functions as an adjuvant and induces tumor antigen-specific immune response via the activation of DCs. Furthermore, FimH has been previously reported to enhance anti-cancer immunity against melanoma and carcinoma in mice (21). However, the mechanism by which FimH contributes to the alteration of the TME, especially via the modulation of TAM functions in solid tumor, remains unclear. In this study, we investigated the hypothesis that FimH promotes the activation of M1 macrophages, as well as the polarization from M2 to M1 macrophages, contributing to the overall antitumor effect.

## 2 Materials and methods

### 2.1 Mice

Female C57BL/6 mice (6–8 weeks old) were obtained from the Shanghai Public Health Clinical Center (SPHCC, Shanghai, China). TLR4-knockout (KO) and B6.129P2-Ly96-KO (MD2-KO) mice were provided by SPHCC. The mice were maintained in the Laboratory Animal Center of SPHCC under 50–60% humidity and at 20–22°C. This study was approved by the Ethics of Animal Experiments Committee of SPHCC (2021-A040-01).

### 2.2 Ethics statement

This study was conducted in accordance with the Declaration of Helsinki and approved by the Institutional Review Board at SPHCC (IRB no. 2021-S044-02). Peripheral blood was provided from healthy donors and peripheral blood mononuclear cells (PBMCs) were obtained from the blood at SPHCC. All donors were informed sufficiently and consented to the study.

### 2.3 Purification of recombinant FimH protein

Recombinant FimH protein was prepared from *E. coli* as previously described (21). Briefly, pET28a-FimH plasmids were constructed and transformed into BL21 competent cells. After incubation in Luria-Bertani (LB) medium containing isopropyl-beta-D-1-thiogalactopyranoside for induced expression, the competent cells were harvested and disrupted by ultrasonication. The target FimH protein was purified from the cell supernatant using a Ni column, and then renatured in a dialysis bag. The endotoxin contaminated in the FimH protein was removed using endotoxin removal resin (Thermo Fisher Scientific, Waltham, MA, USA) according to the manufacturer’s instructions and the endotoxin level in the FimH protein was less than 1 EU/μg detected by Gel Clot Endotoxin Assay Kit (Genscript, Nanjing, China). Lastly, the FimH protein was lyophilized and maintained in −80°C until use.

### 2.4 Reagents and antibodies

LPS (O111:B4) was purchased from Sigma-Aldrich (St. Louis, MO, USA). The following fluorescence-conjugated antibodies were provided from BioLegend (San Diego, CA, USA) and were used for flow cytometry analysis: anti-F4/80 (BM8), anti-CD11b (M1/70), anit-CD206 (C068C2), anti-CD80 (16-10A1), anti-CD86 (GL-1), anti-MHC class II (M5/114.15.2), anti-CD3 (17A2), anti-CD4 (RM4-5), and anti-CD8a (53-6.7).

### 2.5 Preparation and differentiation of murine bone marrow-derived macrophages and peritoneal macrophages

For BMDM, bone marrow was collected by flushing the femurs and tibia of C57BL/6 mice (6–8 weeks old) with cold PBS. After collection, red blood cells (RBC) were lysed with RBC lysis buffer (Thermo Fisher Scientific, Waltham, MA, USA), and the remaining cells were washed twice with cold PBS. To induce macrophage differentiation, bone marrow cells were cultured in RPMI 1640 supplemented with 10% fetal bovine serum (FBS) and 20 ng/mL murine macrophage colony-stimulating factor (M-CSF) (PeproTech, USA) for 6 days. Fresh medium with M-CSF was added every 3 days. After polarization, the cells were phenotyped and used in different assays. For pMAC, C57BL/6 mice were intraperitoneally injected with 1 mL of 3% thioglycollate medium. After 3 days, pMAC were isolated from the peritoneal cavity of the injected C57BL/6 mice by flushing the peritoneal cavity with 5 mL of ice-cold PBS. The cells were centrifuged and resuspended in RPMI-1640 (12633; Gibco) supplemented with 10% FBS for further assays. For M2-like polarization, BMDM on day 7 or pMAC were treated with 20 ng/mL murine IL-4 and 20 ng/mL IL-13 (PeproTech, USA) for 24 h.

### 2.6 Preparation and polarization of human PBMC-derived macrophages

Human PBMCs were collected from the venous blood of healthy volunteers, and in leukocyte reduction chambers, separated via ficoll density gradient (Sigma, St. Louis, MO, USA). CD14^+^ monocytes were positively selected to >95% purity by magnetic activated cell sorting using anti-CD14 microbeads (Miltenyi, USA). For the induction of macrophage differentiation, the sorted CD14^+^ monocytes were cultured in RPMI 1640 supplemented with 10% FBS and 20 ng/mL human M-CSF (PeproTech, USA). Fresh medium with M-CSF was added every 3 days. On day 7, M2-like polarization was achieved by treatment with human 20 ng/mL IL-4 and 20 ng/mL IL-13 (PeproTech, USA) for 24 h. For M0, only PRMI 1640 + 10% FBS was added.

### 2.7 Flow cytometry analysis

The cells were incubated with the unlabeled isotype control antibodies (Abs) and Fc-block Abs for 15 min (BioLegend, San Diego, CA, USA). Then, fluorescence-conjugated Abs was added before incubating the cells on ice for further 30 min. After washing with PBS, the cells were analyzed on FACS Fortessa (Becton Dickinson, Franklin Lakes, New Jersey, USA) using FlowJo v10 software (Tree Star, San Diego, CA, US). Cellular debris and dead cells were excluded by forward- and side-scatter gating and 4’,6-diamidino-2-phenylindole (DAPI) (Sigma-Aldrich, St. Louis, Missouri, US) staining.

### 2.8 Mouse tumor model and TAM isolation

Murine melanoma cell lines B16F10 (CRL-6475; ATCC) were cultured in RPMI 1640 (2 mM glutamine, 100 μg/mL streptomycin, 10% FBS, 100 U/mL penicillin, and 1 M HEPES). Prior to the use, the cells with 70-80% confluence were detached with trypsin-EDTA (0.25%) and washed with PBS twice. For the melanoma models, C57BL/6 mice were subcutaneously (*s.c.*) inoculated with 1 × 10^6^ cells of B16F10. After 7 days of tumor-cell injection, the mice were further divided in three groups and *s.c.* injected with PBS, FimH (2.5 mg/kg), or LPS (1 mg/kg) at the distance of the tumor (21). Total tumors were excised in a sterile dish containing 5 mL of RPMI-1640 media at room temperature (RT) and minced into small pieces using fine scissors. After filtering through a 70-μm cell strainer, the cell suspension was mixed with 10 mL of Ficoll-Paque media and then gently layered on 10 mL of fresh Ficoll-Paque media and centrifuged (1025 × *g*, 20 min, 20°C) with slow acceleration and the brakes turned off (23). The lymphomononuclear layer was collected and cultured for 40 min at 37°C. After three washes, the adherent cells remaining in the plate were detached by using Accutase^®^ Cell Detachment Solution (BioLegend, San Diego, California, USA) and washed for further analysis.

### 2.9 Intracellular cytokine staining

The harvested TAMs were stimulated *in vitro* for 4 h with phorbol 12-myristate 13-acetate (PMA; 50 ng/mL) and ionomycin (1 μM) (Merck, Kenilworth, New Jersey, USA), with the addition of monensin solution (BioLegend, San Diego, California, USA) during the final 2 h. For intracellular cytokine staining, the cells were initially stained to assess surface molecules in the dark for 20 min at RT, then fixed and permeabilized with Cytofix/Cytoperm buffer (eBioscience, San Diego, California, USA) for 20 min at RT, and subsequently incubated with anti-cytokine Abs in Perm/Wash buffer (eBioscience, San Diego, California, USA) for 30 min in the dark at RT. Staining of isotype control IgGs was performed in all experiment.

### 2.10 Immunofluorescence staining

Mice were sacrificed on day 15 after tumor inoculation. The entire tumors were fixed with 4% paraformaldehyde and then embedded in paraffin. Sections of 5 µm in size were cut from the embedding tissue. The sections were further deparaffinized and rehydrated through xylene and graded ethanol series, and subjected to heat treatment in citrate buffer, followed by quenching of endogenous peroxidase activity using 0.3% H_2_O_2_ in methanol. Sections were blocked for 1 h with Fc Receptor Blocker and incubated separately with CD206 antibody (ab300621, 1:1000; Abcam) and F4/80 antibody (70076, 1:500; Cell Signaling Technology). Multiplexed immunofluorescence staining was then performed using Opal 7-color Manual IHC Kit (Akoya, USA). After washing, the stained slides were stained with DAPI for 2 min and scanned by TissueFAXS 200 (TissueGnostics). The acquired images were analyzed by Strata Quest software to assess the number of F4/80^+^ and CD206^+^.

### 2.11 Real-time polymerase chain reaction

Total RNA was extracted from cells using RNAprep Pure Cell/Bacteria Kit (TiangenBiotech, Beijing, China), and reverse-transcribed into complimentary DNA (cDNA) by Oligo (dT) and M-MLV reverse transcriptase (Promega, Madison, Wisconsin, USA). The cDNA was subjected to real-time PCR (Qiagen, Hilden, Germany) for 40 cycles with an annealing and extension temperature of 60 °C on a real-time PCR system (Roche, Basel, Switzerland). The sequences of the primers used were as follows: mouse β-actin: forward, 5′-AGAGGGAAATCGTGCGTGACATCAA-3′, reverse, 5′-ATACCCAAGAAGGAAGGCTGGAAAA-3′; iNOS: forward, 5′-CAGCTGGGCTGTACAAACCTT-3′, reverse, 5′-ATGTGATGTTTGCTTCGGACA-3′; IL-6: forward, 5′-CAAAGCCAGAGTCCTTCAGAG-3′, reverse, 5′-GCCACTCCTTCTGTGACTCC-3′; IL-12p40: forward, 5′-CACATCTGCTGCTCCACAAG-3′, reverse, 5′-CCGTCCGGAGTAATTTGGTG-3′; Arg-1: forward, 5′-GCTCAGGTGAATCGGCCTTTT-3′, reverse, 5′-TGGCTTGCGAGACGTAGAC-3′; CD206: forward, 5′-CTCTGTTCAGCTATTGGACGC-3′, reverse, 5′-CGGAATTTCTGGGATTCAGCTTC-3′; IL-10: forward, 5′-GCCCTTTGCTATGGTGTCCTTTC-3′, reverse, 5′-TCCCTGGTTTCTCTTCCCAAGAC-3′.

### 2.12 Enzyme-linked immunosorbent assay

The concentrations of IL-6, IL-12p70, and TNF-α in the culture medium were measured in triplicate using ELISA kits per the manufacturer’s instructions (BioLegend, San Diego, CA, USA). The detection limit for IL-6 ELISA kit was 2 pg/mL, while the expected minimum detectable concentration of IL-12p70 and TNF-α for this set was 4 pg/mL.

### 2.13 Nitrite assay

The nitrite concentration in the culture medium of BMDM and pMAC was measured in triplicate using an ELISA plate reader at 540 nm absorbance per the manufacturer’s instructions (Beyotime, Shanghai, China).

### 2.14 *In vitro* T cell activation and co-culture

Mouse CD8 T Cell Isolation Kit (BioLegend, San Diego, CA, USA) was used for isolation of CD8 T cells from C57BL/6 mice. For T-cell activation, anti-CD3ϵ (5 μg/mL) (BioLegend, San Diego, CA, USA) was pre-coated in 24 well plates overnight at 4°C. Anti-CD28 (0.5 μg/mL) (BioLegend, San Diego, CA, USA) was added subsequently to plates. For co-culture assay, BMDM at indicated ratios were added to the medium after T cell activation with a ratio of 1:10 for 3 days.

### 2.15 Construction of recombined lentivirus vector and transfection

The CDS sequence of murine TLR4 was downloaded from GenBank (accession no. AF177767.1) and constructed to pLVX-IRES-ZsGreen vector to generate the pLVX-IRES-TLR4 plasmid. After confirmed by sequencing, the pLVX-TLR4 plasmid was then packaged recombined lentivirus. The empty pLVX-IRES-ZsGreen vector was employed as negative control. BMDM were obtained fromTLR4-KO mice and transfected with TLR4 recombined lentivirus. The GFP positive cells were gated as TLR4-restored BMDM.

### 2.16 Statistical analysis

Data were expressed as the mean ± standard error of the mean (SEM). One- or two-way analysis of variance (ANOVA) followed by Tukey’s multiple comparison test and Mann-Whitney U-test were used for the analysis of datasets with the help of SPSS software (IBM, Armonk, NY, USA). P < 0.05 was considered to be statistically significant.

## 3 Results

### 3.1 FimH polarizes M1 macrophages *in vitro*

**Figure 1:**
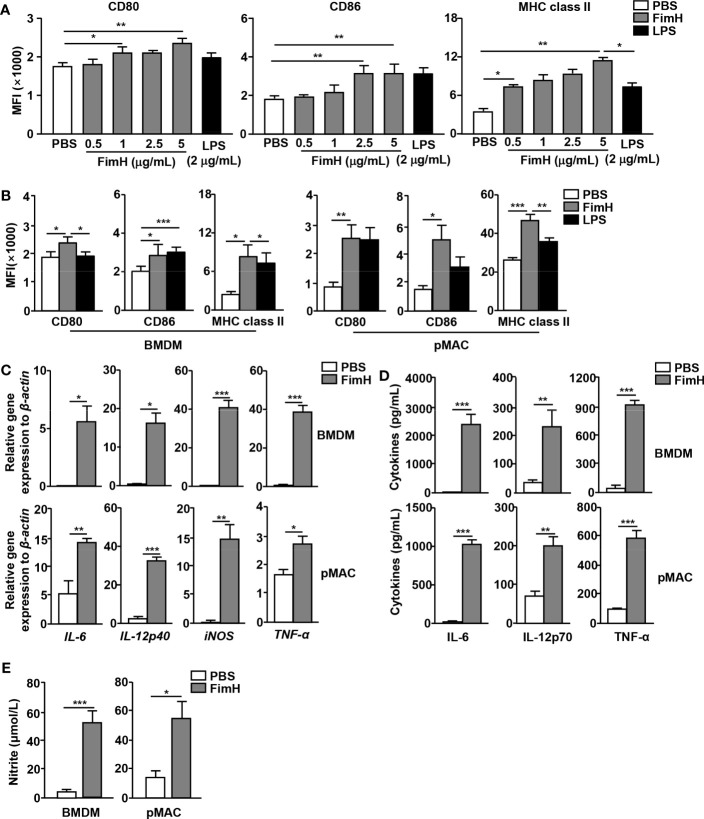
Macrophage activation by FimH treatment. Bone marrow cells were incubated with 20 ng/mL murine macrophage colony-stimulating factor (M-CSF) for 6 days to generate bone marrow-derived macrophages (BMDM) and then stimulated with 0.5, 1, 2.5, and 5 μg/mL FimH or LPS for 24 h. **(A)** Mean fluorescence intensity (MFI) of CD80, CD86, and major histocompatibility complex (MHC) class II in BMDM. BMDM and peritoneal macrophage (pMAC) were stimulated with FimH (5 μg/mL) or LPS (2 μg/mL) for 24 h. **(B)** MFI of CD80, CD86, and MHC class II in BMDM (left panel) and pMAC (right panel). **(C)** Mean values of the mRNA expression levels in the BMDM (upper panel) and pMAC (lower panel). **(D)** Concentrations of interleukin (IL)-6, IL-12, and tumor necrosis factor alpha (TNF-α) in the culture medium of BMDM (upper panel) and pMAC (lower panel). **(E)** Concentration of nitrite in culture medium of BMDM (left panel) and pMAC (right panel). (^*^p < 0.05, ^**^p < 0.01, ^***^p < 0.001).

To evaluate the potential of FimH on macrophages polarization, murine BMDM and pMAC were treated with FimH. Treatment with FimH promoted dose-dependent increases in the expression levels of the M1 macrophage markers CD80, CD86, and MHC class II in BMDM (**Figure 1A**). In addition, FimH also induced the upregulation of CD80, CD86, and MHC class II in both BMDM and pMAC (**Figure 1B**). Since M1 macrophages released pro-inflammatory cytokines, cytokine production in the macrophages was further examined. Treatment with FimH induced a notable upregulation of the IL-6, IL-12p40, iNOS, and TNF-α mRNA levels in BMDM and pMAC (**Figure 1C**). The secretion levels of IL-6, IL-12p70, and TNF-α, as well as nitrite, also increased substantially in the culture medium of BMDM and pMAC after FimH treatment (**Figures 1D, E**). These results indicated that FimH could induce the activation of M1 macrophages in mice *in vitro*.

### 3.2 FimH reverses M2 macrophages toward an M1 profile *in vitro*

**Figure 2:**
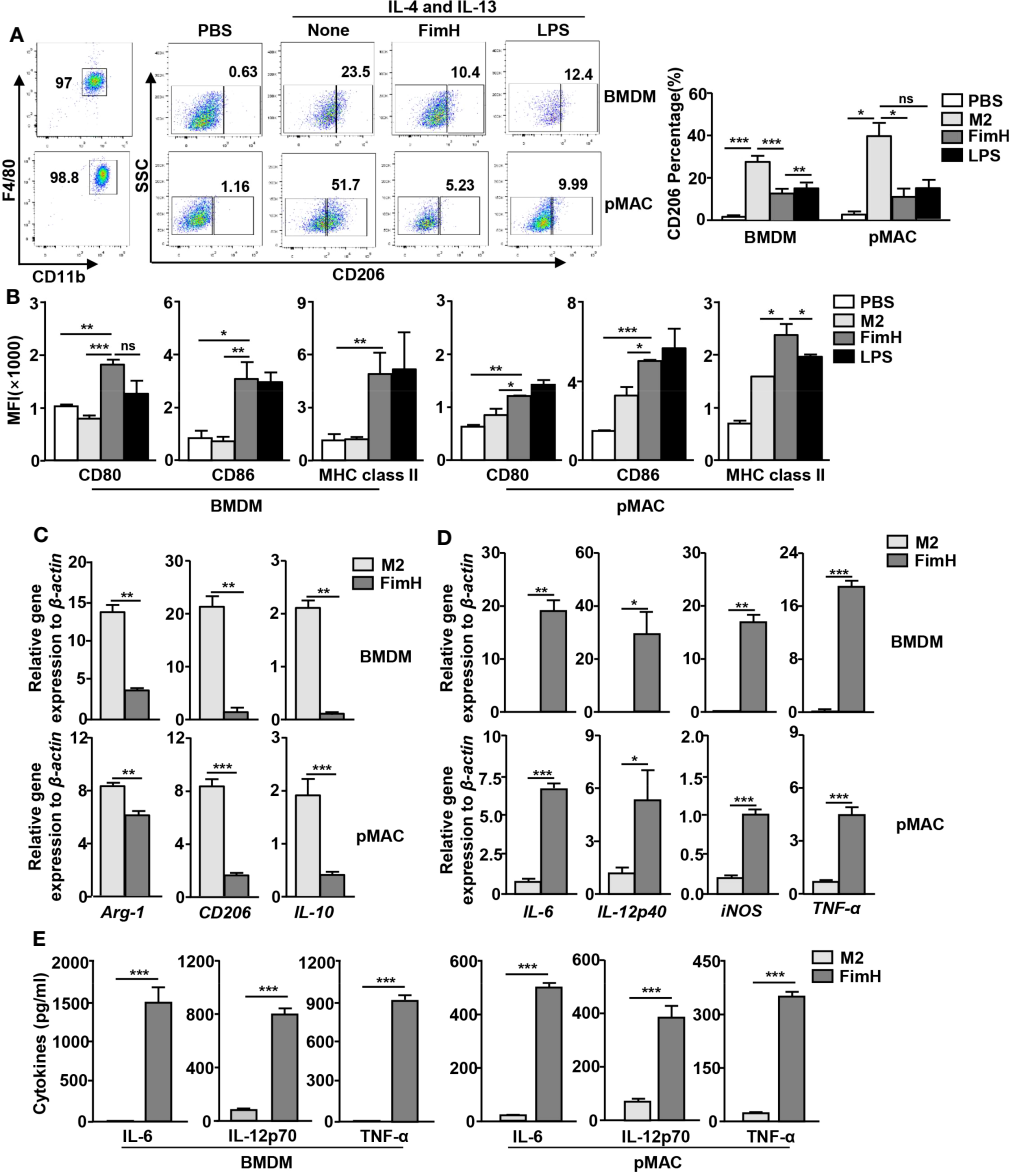
FimH reverses M2 macrophages toward an M1 profile. Bone marrow-derived macrophages (BMDM) and peritoneal macrophage (pMAC) were obtained from C57BL/6 mice and treated with 20 ng/mL murine interleukin (IL)-4 and 20 ng/mL IL-13 for 24 h, and then stimulated with FimH (5 μg/mL) or LPS (2 μg/mL) for an additional 24 h. **(A)** Representative flow cytometry gating of macrophages (left panel) and quantification of the CD206^+^ macrophages (right panel). **(B)** Mean fluorescence intensity (MFI) of CD80, CD86, and major histocompatibility complex (MHC) class II in BMDM (left panel) and pMAC (right panel). **(C, D)** Mean values of the mRNA expression levels in the BMDM (upper panel) and pMAC (lower panel). **(E)** Concentrations of IL-6, IL-12, and tumor necrosis factor alpha (TNF-α) in the culture medium of BMDM (left panel) and pMAC (right panel). (^*^p < 0.05, ^**^p < 0.01, ^***^p < 0.001, n.s., none significant).

Based on the findings that FimH effectively drove mouse BMDM and pMAC toward the M1 phenotype, we further examined whether FimH could reverse M2 to M1 macrophages. For the induction of M2 macrophages, mouse BMDM and pMAC were incubated with murine IL-4 and IL-13 for 24 h, and the cells were stimulated with FimH or LPS for another 24 h. The upregulated expression levels of CD206, the M2 macrophage specific marker, by IL-4 and IL-13 treatment in BMDM and pMAC decreased on FimH administration (**Figure 2A**). Even during the differentiation condition of M2 macrophages, the expression of M1-specific surface markers increased after treatment with FimH (**Figure 2B**). Furthermore, the mRNA levels of M2 macrophage activation markers decreased on FimH administration (**Figure 2C**). In contrast, the mRNA and protein levels of M1 macrophage markers increased substantially on FimH administration (**Figures 2D, E**). In addition, failure of IFN-γ production in CD8 T cells by co-culturing with M2 macrophages was reversed in FimH treated macrophages (**Figure S1**). These results suggested that FimH promoted M2-to-M1 polarization.

### 3.3 Polarity of FimH-induced M1 macrophages is TLR4 dependent

**Figure 3:**
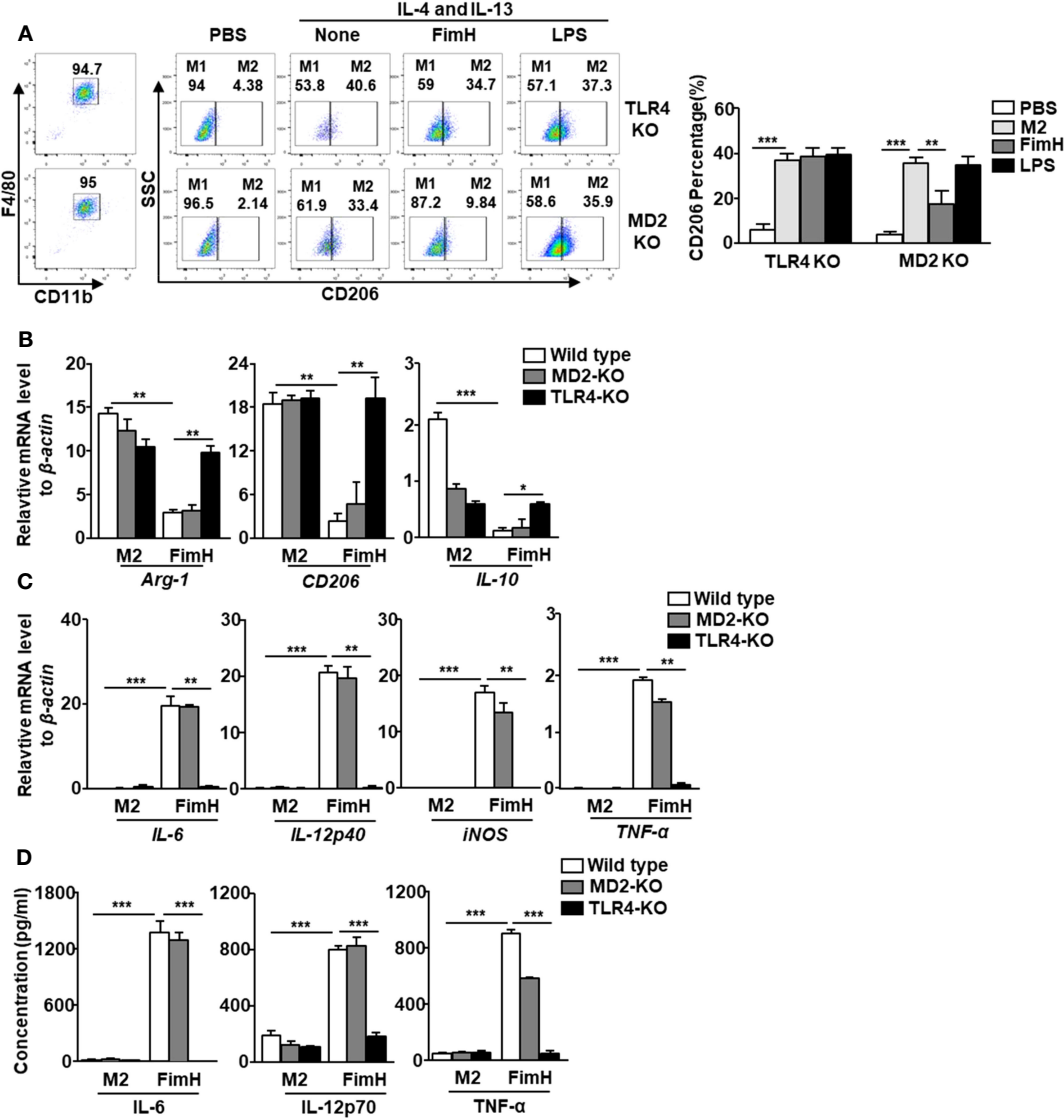
FimH reprograms macrophages in TLR4-dependent manner. Bone marrow-derived macrophages (BMDM) were obtained from toll-like receptor 4 (TLR4)-knockout (KO) and myeloid differentiation protein 2 (MD2)-KO mice and treated with 20 ng/mL murine interleukin (IL)-4 and 20 ng/mL IL-13 for 24 h, and then stimulated with FimH (5 μg/mL) or LPS (2 μg/mL) for an additional 24 h. **(A)** Representative flow cytometry gating of macrophages (left panel) and quantification of the CD206^+^ macrophages (right panel). **(B)** Mean values of the mRNA expression levels for M2 profile in the BMDM. **(C)** Mean values of the mRNA expression levels for M1 profile in the BMDM. **(D)** Concentrations of IL-6, IL-12, and tumor necrosis factor alpha (TNF-α) in the culture medium of BMDM. (^*^p < 0.05, ^**^p < 0.01, ^***^p < 0.001).

In a previous research, we showed that FimH promoted the activation of DCs in a TLR4-dependent manner (21). Since FimH promoted M2 macrophages towards M1 polarization, we further examined whether the action of M1 polarization by FimH was also dependent on TLR4 signaling pathway. The upregulated expression of CD206 in BMDM by IL-4 and IL-13 treatment was preserved in the TLR4-KO BMDM treated with FimH, while it decreased by FimH treatment in MD2-KO BMDM (**Figure 3A**). To further confirm the dependence of TLR4 signaling pathway, TLR4 was constructed to pLVX-IRES-ZsGreen vector and re-introduced to TLR4-KO BMDM by lentivirus transfection (**Figure S2A**). The preserved expression of CD206 in control lentivirus transfected TLR4-KO BMDM was decreased again by FimH treatment in TLR4-restored TLR4 KO BMDM (**Figure S2B**). Moreover, the mRNA levels of M2 macrophage markers, including Arg-1, CD206, and IL-10, were also not downregulated by FimH in TLR4-KO BMDM, whereas it decreased in the MD2-KO BMDM on FimH treatment (**Figure 3B**). Meanwhile, FimH treatment failed to promote the conversion of M1 macrophages from M2 macrophages, as indicated by the fact that FimH treatment did not upregulate the mRNA levels of IL-6, IL-12p40, iNOS, and TNF-α in TLR4-KO BMDM, while MD2-KO BMDM upregulated the mRNA levels on FimH treatment (**Figure 3C**). Consistent with the mRNA levels, the production levels of IL-6, IL-12p70, and TNF-α did not increase in the TLR4-KO BMDM by FimH, whereas FimH induced a notable upregulation of IL-6, IL-12p70, and TNF-α in MD2-KO BMDM (**Figure 3D**). These results indicated that FimH-mediated M1 polarization was dependent on TLR4, but not MD2.

### 3.4 FimH converts TAMs into M1 macrophages in mice *in vivo*

**Figure 4:**
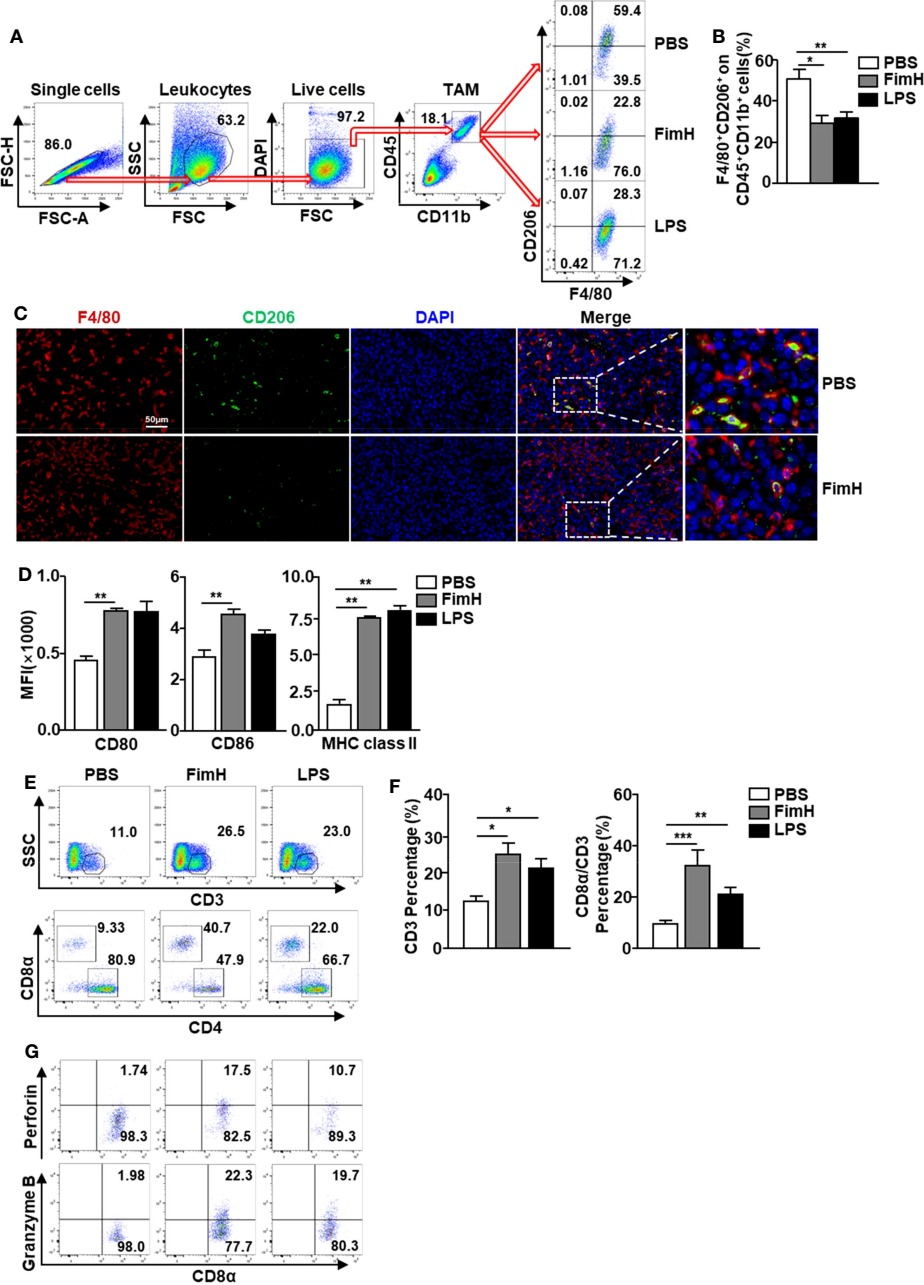
FimH promotes TAM polarization to M1 status. C57BL/6 mice were subcutaneously (*s.c.*) inoculated with 1 × 10^6^ B16F10 cells. After 7 days of tumor-cell injection, the mice were further divided in three groups and *s.c.* injected with PBS, FimH (2.5 mg/Kg), or LPS (1 mg/Kg). **(A)** Representative flow cytometry gating of tumor-associated macrophage (TAM) in tumor. **(B)** Percentage of CD206^+^ F4/80^+^ macrophages. **(C)** Immunofluorescence detection of TAMs in the tumor sections by sequential staining with anti-F4/80 and anti-CD206 antibodies, and nuclei counterstained by DAPI. Red and green signals indicate the detected F4/80^+^ and CD206^+^ cells, respectively. Magnification: 200×. **(D)** Mean fluorescence intensity (MFI) of CD80, CD86, and MHC class II in TAM. **(E)** Tumor infiltration T cells were measured in the mouse tumor *in vivo*. Dot plots showed the percentages of CD3^+^ and CD8α^+^ T cells. **(F)** Mean percentages of CD3 (left panel) and CD8α (right panel) cells. **(G)** Intracellular perforin and granzyme B producing CD8α^+^ T cells in the tumor. (n = 6 mice, ^*^p < 0.05, ^**^p < 0.01, ^***^p < 0.001).

Based on the fact that FimH promoted M1 polarization from M2 macrophages, we further examined the effect of FimH on the polarization of M1 macrophages from TAMs in tumor-bearing mice. To achieve this, C57BL/6 mice were injected *s.c.* with 1 × 10^6^ B16F10 cells. Seven days after tumor-cell inoculated, the mice were administered a *s.c.* injection of PBS, 2.5 mg/Kg FimH, or 1 mg/Kg LPS three times (3 days apart). Although not significant, the size and weight of tumors decreased considerably on FimH treatment (**Figure S3**). As shown in **Figure 4A**, we defined TAMs in the tumor and found that the expression of CD206 in TAMs decreased notably by FimH compared with the PBS control (**Figure 4B**). A reduction in CD206 was also confirmed by the immunofluorescence staining in B16 tumor tissue (**Figure 4C**). Moreover, FimH promoted the upregulation of co-stimulator and MHC class II expression in TAM, which indicated that the TAMs were converted to M1 macrophages (**Figure 4D**). As M1 macrophages promote T cell activation, we next examined the effect of FimH-activated M1 macrophages on the induction of T cell activation in tumor. The frequencies and percentages of both CD3^+^ and CD8^+^ T cells in tumor increased notably on FimH treatment (**Figures 4E, F**). In addition, the intracellular levels of perforin and granzyme B, the cytotoxic marker, in CD8^+^ T cells also increased substantially on FimH treatment (**Figure 4G**). These results suggested that FimH could polarize TAMs to M1 macrophages.

### 3.5 FimH promotes human monocyte-derived macrophages toward the M1 phenotype

**Figure 5:**
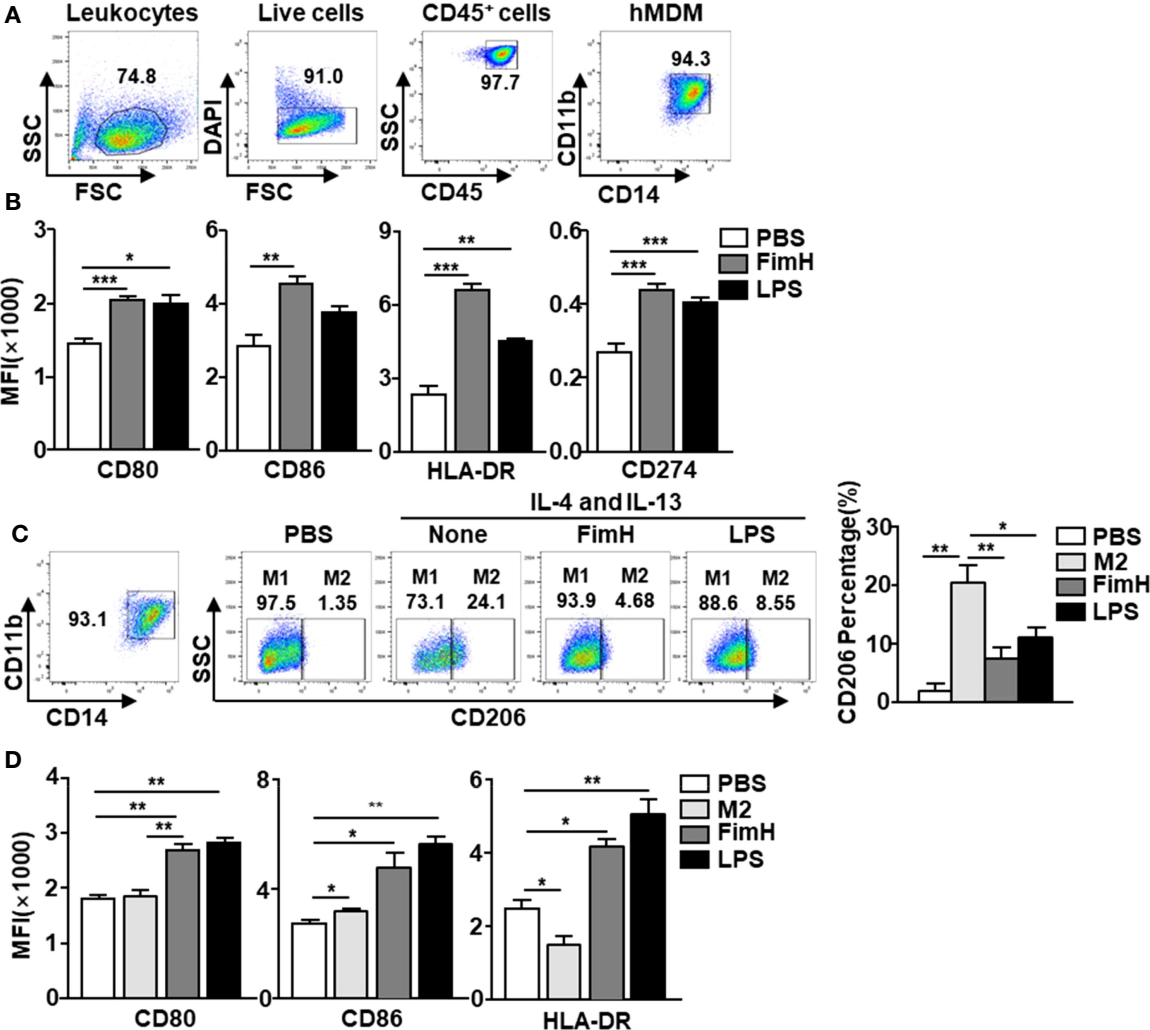
FimH promotes human monocyte-derived macrophages (hMDM) toward M1 phenotype. Peripheral blood mononuclear cells (PBMCs) were collected from healthy volunteers. The CD14^+^ monocytes from PBMCs were positively selected and cultured with 20 ng/mL human M-CSF for 7 days to derive macrophages. The hMDM were then stimulated with FimH (5 μg/mL) or LPS (2 μg/mL) for 24 h. **(A)** Gating of hMDM by flow cytometry. **(B)** Mean fluorescence intensity (MFI) of CD80, CD86, HLA-DR, and CD274 in hMDM. M2-like polarization was achieved by treatment with human 20 ng/mL IL-4 and 20 ng/mL IL-13 on day 7 for 24 h. **(C)** Representative flow cytometry gating of hMDM (left panel) and quantification of the CD206^+^ macrophages (right panel). **(D)** MFI of CD80, CD86, and HLA-DR in hMDM. (^*^p < 0.05, ^**^p < 0.01, ^***^p < 0.001).

Since FimH promoted M1 macrophage polarization in mice, we next examined the effects of FimH on human macrophages. To achieve this, human monocyte-derived macrophages (hMDM) were generated from peripheral blood monocytes by M-CSF (**Figure 5A**) and the cells were treated with FimH and LPS. The co-stimulator and MHC class II expression levels in hMDM were markedly upregulated by FimH compared with those treated with PBS (**Figure 5B**). Thereafter, we also examined the conversion of M1 macrophages from M2 macrophage in hMDM and found that the upregulated CD206 expression levels in M2 hMDM were markedly reduced by FimH treatment (**Figure 5C**). In addition, the expression of M1 macrophage makers also increased notably by FimH in IL-4 and IL-13-treated hMDM (**Figure 5D**). These results suggested that FimH could promote the polarization of human M1 macrophages from M2 macrophages.

### 3.6 FimH enhances the anti-cancer immunity of anti-PD-L1 antibody via the induction of M1 polarization from TAMs

**Figure 6:**
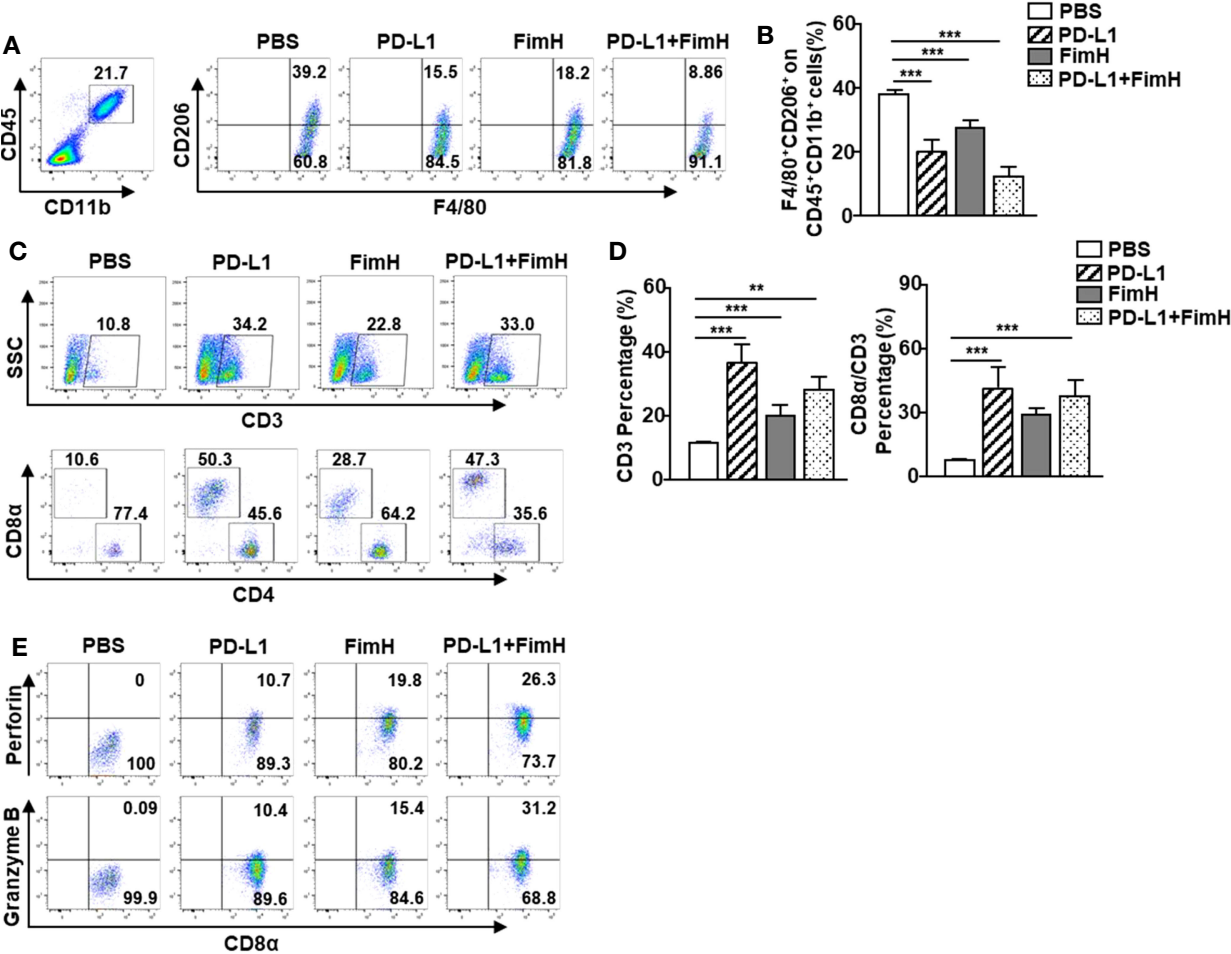
FimH enhances anti-cancer immunity of anti-PD-L1 antibody via the induction of M1 polarization from TAM. C57BL/6 mice were subcutaneously (*s.c.*) injected with 1 × 10^6^ B16F10 cells. After 5 days of tumor-cell injection, the mice were treated with anti-PD-L1 Abs (10 mg/Kg), FimH (2.5 mg/Kg) or a combination of anti-PD-L1 Abs and FimH for every 5 days. **(A)** Representative flow cytometry gating of tumor-associated macrophage (TAM) in tumor. **(B)** Mean percentage of CD206^+^ F4/80^+^ macrophages. **(C)** Representative flow cytometry dot plots of CD3^+^ and CD8α^+^ T cells in the mouse tumor. **(D)** Mean percentages of CD3 (left panel) and CD8α (right panel) cells. **(E)** Intracellular perforin and granzyme B producing CD8α^+^ T cells in the tumor. (n = 6 mice, ^**^p < 0.01, ^***^p < 0.001).

Since FimH could induce the conversion of TAMs to M1 macrophages, we further examined whether FimH-induced M1 polarization could enhance the anti-tumor activity of the anti-PD-L1 antibody. The M2 macrophages, induced by murine IL-4 and IL-13 from mouse BMDM, were reversed to the M1 phenotype with an upregulated expression of M1-specific surface markers by a combination of anti-PD-L1 Abs and FimH (**Figure S4**). C57BL/6 mice were *s.c.* injected with 1 × 10^6^ B16F10 cells. After 5 days of tumor cell injection, the mice were treated with anti-PD-L1 Abs (10 mg/Kg), FimH (2.5 mg/Kg), or a combination of anti-PD-L1 Abs and FimH every 5 days until 15 days after tumor injection. Both anti-PD-L1 and FimH treatment notably decreased the expression of CD206 in the TAMs compared with the PBS control, while the combined treatment enhanced the effect of reversing TAMs in tumors (**Figures 6A, B**). Moreover, the frequencies and percentages of both CD3^+^ and CD8^+^ T cells in tumors increased remarkably after the combined treatment of anti-PD-L1 Abs and FimH (**Figures 6C, D**). Furthermore, the intracellular levels of both perforin and granzyme B in tumor infiltrated CD8^+^ T cells increased notably after the combined treatment (**Figure 6E**). These results suggest that FimH could enhance the anti-cancer immunity of anti-PD-L1 antibody via the induction of M1 polarization from TAMs.

## 4 Discussion

Antigen-presenting cells (APC), such as macrophages and DCs, are the first defense immune cells against tumor cells (24). These rely on pattern recognition receptors, and APC detect pathogen-associated molecular pattern to orchestrate downstream immune responses (25). In addition, damage-associated molecular patterns also induce the activation of immune cells, which are produced from dying cells, as well as necrotic tumor cells (26). However, solid tumor cells promote the evasion of the innate immune system-mediated activation of immune responses by building up the immunosuppressive microenvironment (27). The main contributors of immunosuppression in the tumor microenvironment are TAMs, despite a crucial role played by the macrophages in anti-tumor activity by mediating tumoricidal activity and eliciting adaptive immune responses. Therefore, therapeutic strategies targeting macrophages for the induction of M1 polarization from TAMs have been evaluated in clinical trials (28). In this study, we found that FimH reversed M2 macrophages into anti-tumor M1 macrophages both *in vitro* and *in vivo*, as well as enhanced T cell infiltration in tumors, which further contributed to the anti-cancer effect in the TME. These results highlight the role of FimH as a candidate molecule for the alteration of TAMs to M1 macrophages, as well as the induction of anti-tumor immune responses.

It has been reported that TLR ligands play an important role in the polarization of macrophages (29). R848 (TLR7/8 agonist), and lefitolimod (TLR9 agonist) have been found to transform TAMs to M1 macrophages and limit tumor progression (30, 31). Moreover, M2 macrophages polarized by IL-4 and IL-13 can be reshaped into the M1 subtype via the TLR4 signaling pathway after being exposed to LPS (32). However, LPS cannot be used in humans since they have a high sensitivity to endotoxin levels. Although monophosphoryl lipid A (MPLA), derived from modified LPS with removed toxicity can induce innate immune cell activation, it requires a combination of IFN-γ for the induction of M1 macrophage polarization from TAMs (33). In addition, MPLA is not soluble, which requires the formulation with other lipids as liposomes. FimH also stimulates TLR4 and induces the activation of macrophages. More importantly, FimH can promote the repolarization of TAMs into M1 macrophages without the need for any combination treatment. In addition, FimH is a soluble protein that has been reported to have low toxicity in mice *in vivo* (21). Therefore, FimH could represent a new tumor immunotherapeutic agent to overcome the limitations of MPLA.

Previous studies have showed that FimH can elicit antibacterial, antiviral, and immunomodulatory effects (34, 35). The well-defined function of FimH is in the induction of immune cells, such as NK cells and DCs, through the TLR4 signaling pathway (20). As FimH induces the activation of DCs, FimH treatment can be combined with antigens that elicit antigen-specific cytotoxic T cell activation, as well as anti-tumor immunity (21). Although we have shown that FimH combined with antigens can be used in a therapeutic tumor vaccine, this was insufficient to explain how T cells infiltrated solid tumors without altering the immunosuppressive tumor microenvironment. In this study, we found that FimH repolarized TAMs to M1 macrophages, which indicated that FimH altered the immunosuppressive tumor microenvironment to an immune responsive microenvironment. The findings presented in this study provide a basis future studies to elucidate the mechanism underlying the potential anticancer effects of FimH.

The complex interaction between macrophages and the PD-1/PD-L1 signaling pathway may be the key factor for the function of immune checkpoints. Although it is widely assumed that PD-1/PD-L1 and other immune checkpoint Abs eliminate tumors by restoring the immune cytotoxic ability of T cells, treatment with the anti-PD-L1 Abs in T-cell deficiency tumor mice has been shown to inhibit tumor growth (36). Furthermore, studies have shown that PD-L1 signaling can regulate the proliferation and activation of macrophages (37). Anti-PD-L1 treatment leads to the polarization of TAMs towards a more proinflammatory phenotype (38). The expression of PD-L1 in tumor-infiltrating immune cells, particularly TAM, is linked to the clinical response to anti-PD-L1 Abs therapy (39). In a previous study, we found that FimH enhanced the anti-tumor effect of anti-PD-L1 Abs (21). In the present study, we verified that FimH treatment could enhance the remodeling effect of anti-PD-L1 on macrophage polarization in both BMDM and the TME. Moreover, the combination of anti-PD-L1 and FimH was found to increase the percentage and intracellular cytokine expression of infiltrated T cells in the TME. Therefore, the enhancement of the anti-cancer effect by the combination of anti-PD-L1 and FimH could be explained by the remodeled TAMs and the increased tumor infiltration T cells in the TME. Based on these findings, further studies will be needed on the signaling pathway responsible for the synergistic enhancement of the anti-tumor effect via the reshaping of TAMs by FimH and anti-PD-L1 Abs.

To summarize, this study highlighted the potential effect of FimH on the activation of macrophages, responsible for the repolarization of M2 macrophages into the M1 phenotype via the TLR4 signaling pathway. In the TME, FimH could also reprogram TAM polarization to the M1 status, as well as enhance the anti-tumor activity of immune checkpoint blockade. When combined, these findings demonstrated that FimH could be used as an adjuvant for the treatment of tumors by influencing macrophage activity.

## Data availability statement

The original contributions presented in the study are included in the article/**Supplementary Material**. Further inquiries can be directed to the corresponding author.

## Ethics statement

The studies involving humans were approved by This study was conducted in accordance with the Declaration of Helsinki and approved by the Institutional Review Board at Shanghai Public Health Clinical Center (IRB no. 2021-S044-02). The studies were conducted in accordance with the local legislation and institutional requirements. Written informed consent for participation in this study was provided by the participants’ legal guardians/next of kin. The animal study was approved by This study was approved by the Ethics of Animal Experiments Committee of Shanghai Public Health Clinical Center (2021-A040-01). The study was conducted in accordance with the local legislation and institutional requirements.

## Author contributions

J-OJ and WZ designed the experiments and wrote the manuscript. WZ participated in the experiments and data analysis. LX participated in the experiments. J-OJ and WZ reviewed the manuscript. XZ and JX helped with manuscript writing and made important corrections to the manuscript. All authors reviewed and edited the final draft. All authors contributed to the article and approved the submitted version.
