# Supplementary material for: Escherichia coli adhesion portion FimH polarizes M2 macrophages to M1 macrophages in tumor microenvironment via toll-like receptor 4
Source: Front Immunol. 2023 Sep 1;14:1213467. doi: 10.3389/fimmu.2023.1213467 (PMC10502728; doi:10.3389/fimmu.2023.1213467)
Supplement: Supplementary file 1 [file DataSheet_1.docx]

***Supplementary Material***

**Escherichia coli adhesion portion FimH polarizes M2 macrophages to M1 macrophages in tumor microenvironment via toll-like receptor 4**

Wei Zhang^1^, Li Xu^1^, Xiaoyan Zhang^2^, Jianqing Xu^2^, Jun-O Jin^1,3*^

^1^Shanghai Public Health Clinical Center, Shanghai Medical College, Fudan University, Shanghai 201508, China

^2^The Laboratory for Immunotherapy, Clinical Center for BioTherapy, Zhongshan Hospital, Fudan University, Shanghai 200032, China

^3^Dpartment of Microbiology, University of Ulsan College of Medicine, ASAN Medical Center, Seoul, 05505, South Korea

***Correspondence:**

Jun-O Jin

[junojin@amc.seoul.kr](mailto:junojin@amc.seoul.kr)

**
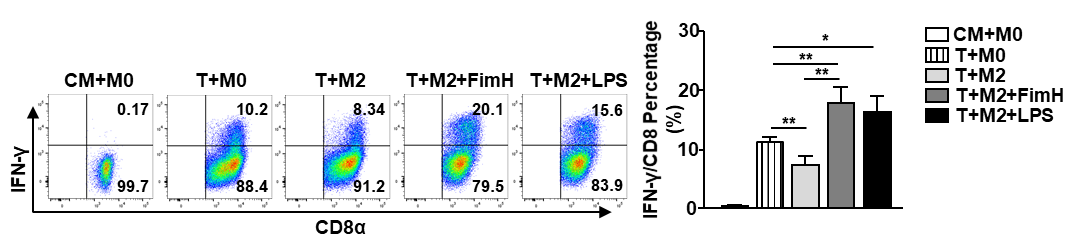
Supplementary Figure 1. FimH reversed IFN-γ production in CD8^+^ T cells**

Bone marrow-derived macrophages (BMDM) were obtained from C57BL/6 mice and treated with 20 ng/mL murine interleukin (IL)-4 and 20 ng/mL IL-13 for 24 h for M2 macrophages. M2 macrophages were further stimulated with FimH (5 μg/mL) or LPS (2 μg/mL) for an additional 24 h. For T-cell activation, anti-CD3ε was pre-coated in 24 well plates overnight at 4 °C. Subsequently anti-CD28 was added to the plates. For co-culture assay, BMDM at indicated ratios were added to the medium after T cell activation for 3 days. CM stands for control medium, which T cells were cultured without CD3/CD28 stimulation. M0 stands for BMDM without stimulation. Intracellular IFN-γ producing T cells (left panel) and mean percentage of the IFN-γ^+^ T cells (right panel). (^*^*p* < 0.05, ^**^*p* < 0.01.)

**
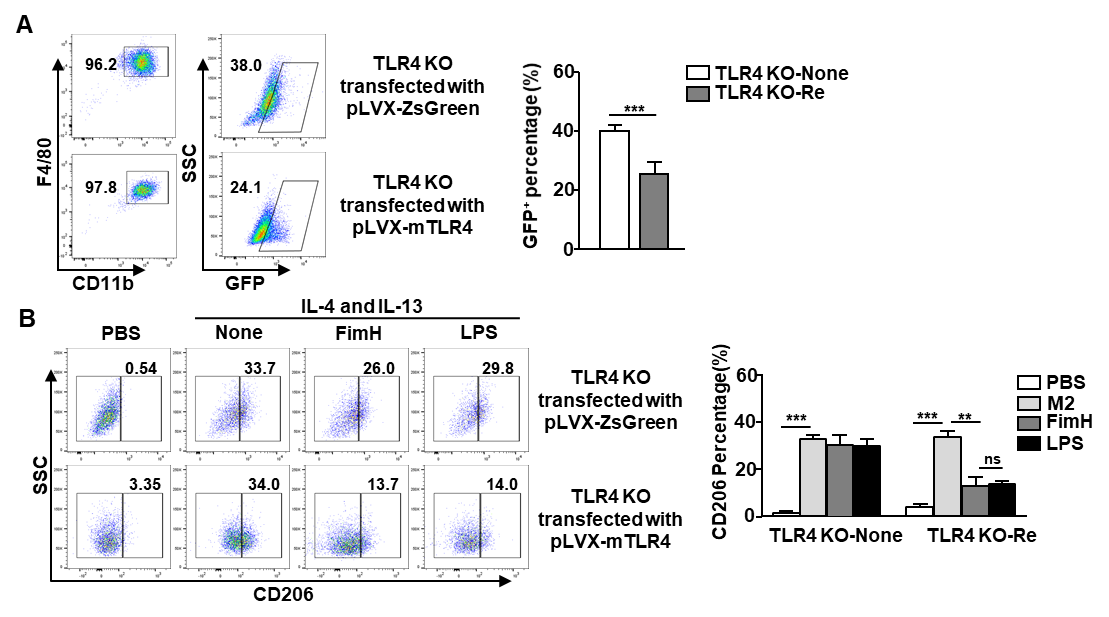
Supplementary Figure 2. FimH-induced M1 macrophages in TLR4-restored BMDM**

The murine TLR4 was constructed to pLVX-IRES-ZsGreen vector to generate the pLVX-TLR4 plasmid, and packaged recombined lentivirus. The empty pLVX-IRES-ZsGreen vector was employed as negative control. BMDM were obtained from TLR4-knockout (KO) mice and transfected with TLR4 recombined lentivirus. The BMDM were then treated with 20 ng/mL murine IL-4 and 20 ng/mL IL-13 for 24 h, and stimulated with FimH (5 μg/mL) or LPS (2 μg/mL) for an additional 24 h. (A) The percentage of the transfection BMDM. (B) Representative flow cytometry gating of macrophages (left panel) and quantification of the CD206^+^ macrophages (right panel). (^**^*p* < 0.01, ^***^*p* < 0.001.)


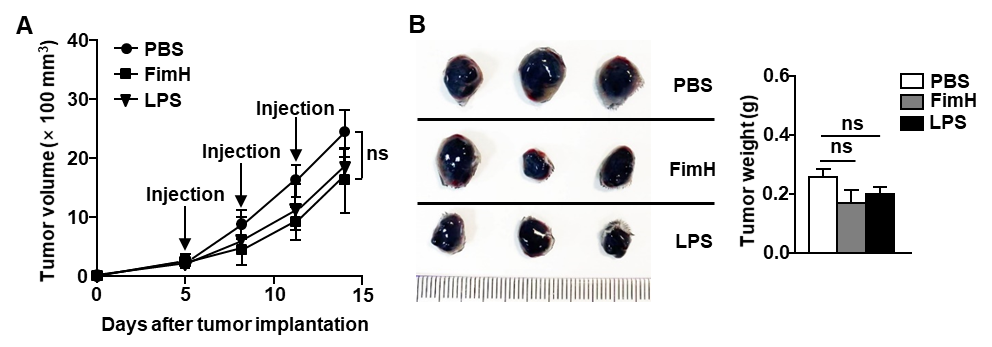


**Supplementary Figure 3.** **Effect of FimH on tumor growth**

C57BL/6 mice were injected with B16F10 melanoma cells and treated with PBS, FimH, and LPS, as shown in Figure 4. (A) Tumor growth curves (n = 6) and (B) Representative tumor mass from treated mice (left panel) and mean tumor weight (right panel).


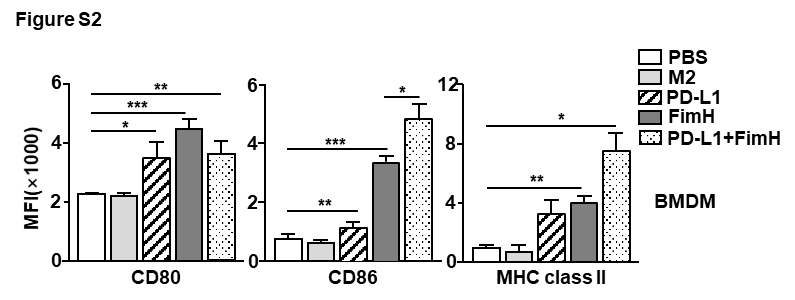


**Supplementary Figure 4.** **M1 polarization from M2 macrophages by an anti-PD-L1 Abs and FimH**

M2 macrophages induced by murine IL-4 and IL-13 were treated with anti-PD-L1 Abs (PD-L1), FimH and the combination of anti-PD-L1 Abs and FimH. The surface expression levels of CD80, CD86, and MHC class II were analyzed by flow cytometry. (^*^*p* < 0.05, ^**^*p* < 0.01, ^***^*p* < 0.001.)
